# Supplementary material for: Association of Antihypertensive Effects of Esaxerenone with the Internal Sodium Balance in Dahl Salt-Sensitive Hypertensive Rats
Source: Int J Mol Sci. 2022 Aug 10;23(16):8915. doi: 10.3390/ijms23168915 (PMC9408866; doi:10.3390/ijms23168915)
Supplement: Supplementary file 1 [file ijms-23-08915-s001.zip › ijms-1816697-supplementary.pdf]

Figure S1

A

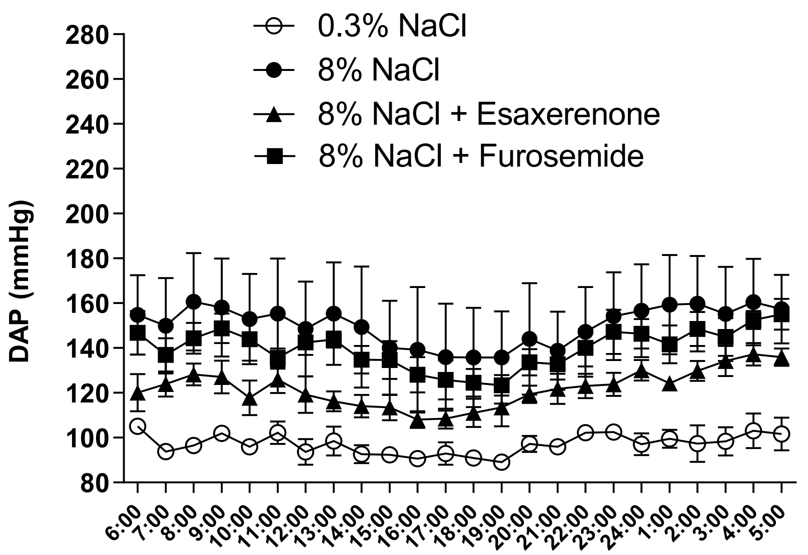

0.3% NaCl
8% NaCl
8% NaCl + Esaxerenone
8% NaCl + Furosemide
DAP (mmHg)
280
260
240
220
200
180
160
140
120
100
80
6:00
7:00
8:00
9:00
10:00
11:00
12:00
13:00
14:00
15:00
16:00
17:00
18:00
19:00
20:00
21:00
22:00
23:00
24:00
1:00
2:00
3:00
4:00
5:00

B

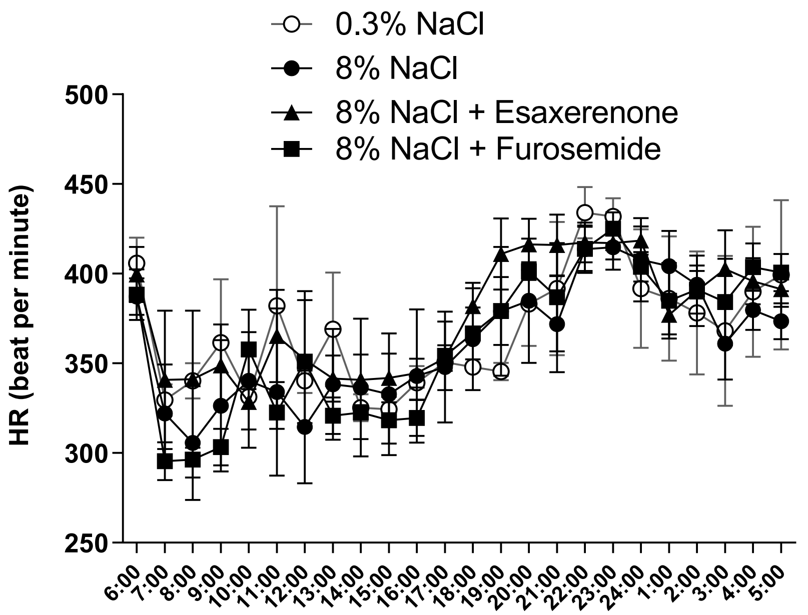

0.3% NaCl
8% NaCl
8% NaCl + Esaxerenone
8% NaCl + Furosemide
HR (beat per minute)
500
450
400
350
300
250
6:00
7:00
8:00
9:00
10:00
11:00
12:00
13:00
14:00
15:00
16:00
17:00
18:00
19:00
20:00
21:00
22:00
23:00
24:00
1:00
2:00
3:00
4:00
5:00

Figure S2

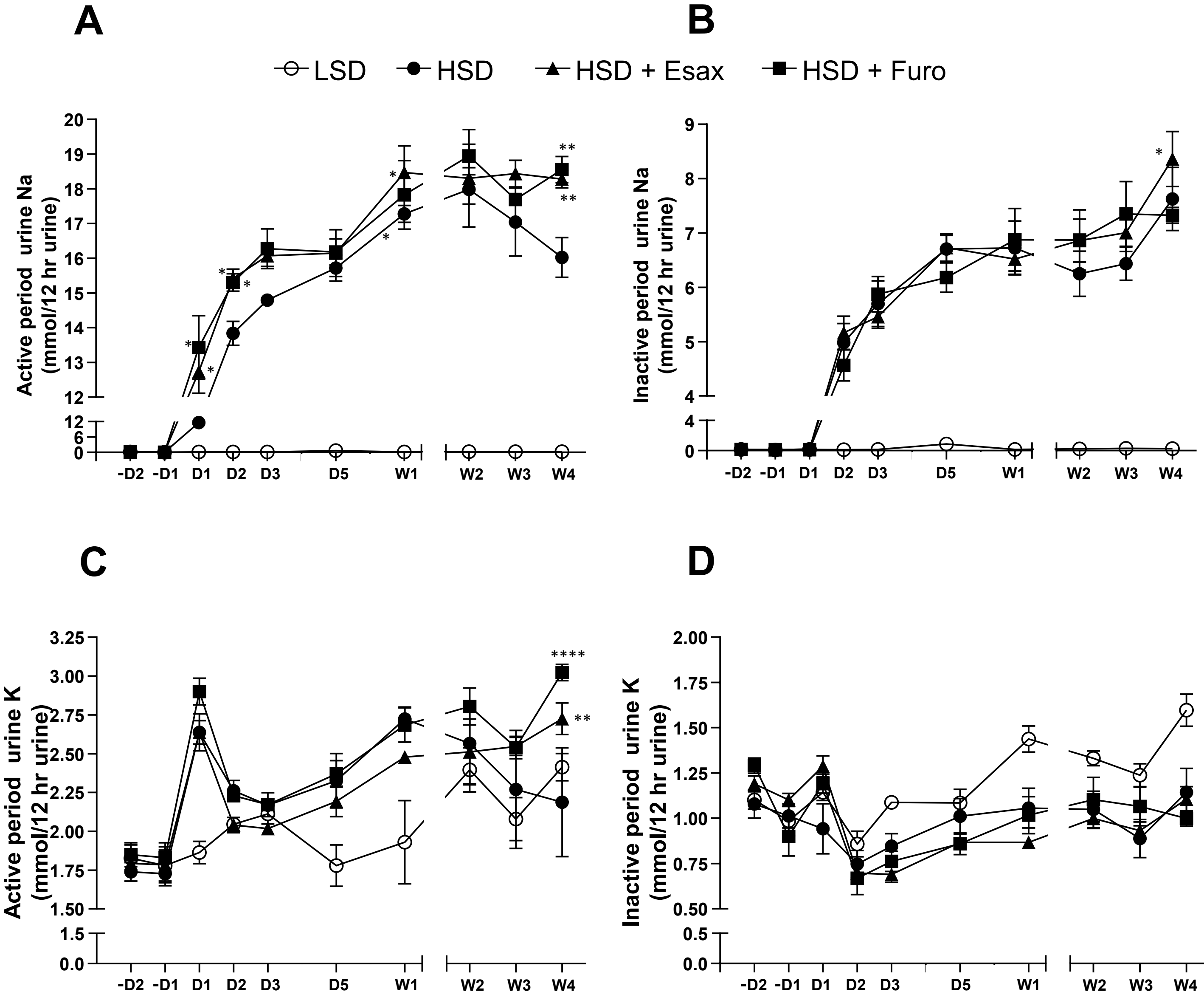
A
B
LSD
HSD
HSD + Esax
HSD + Furo
Active period urine Na (mmol/12 hr urine)
Inactive period urine Na (mmol/12 hr urine)
-D2
-D1
D1
D2
D3
D5
W1
W2
W3
W4
C
D
Active period urine K (mmol/12 hr urine)
Inactive period urine K (mmol/12 hr urine)

Figure S3

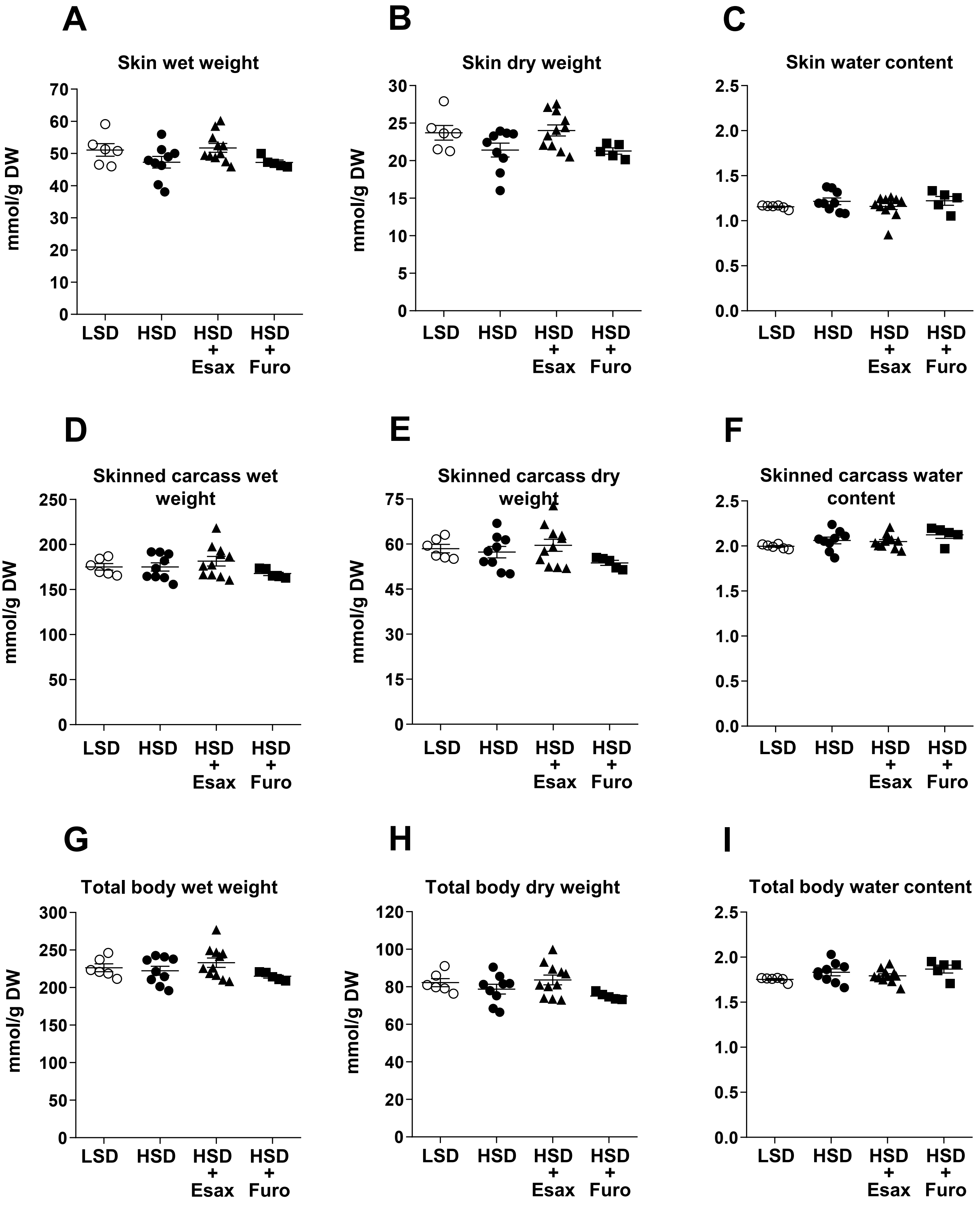
A
Skin wet weight
B
Skin dry weight
C
Skin water content
D
Skinned carcass wet weight
E
Skinned carcass dry weight
F
Skinned carcass water content
G
Total body wet weight
H
Total body dry weight
I
Total body water content
mmol/g DW
LSD
HSD
HSD + Esax
HSD + Furo

Supplementary figure legends

Figure S1. Changes in DAP and heart rate in HS-loaded DSS rats. Twenty-four-hour (A) DAP and (B) heart rate as measured by a radiotelemetry system in conscious rats after 4 weeks of treatment.

Figure S2. Urinary excretion of Na, and K. Urinary excretion of Na in the (A) active and (B) inactive periods. Urinary excretion of K in the (C) active and (D) inactive periods. *p<0.05, **p<0.01, ****p<0.0001 vs an HSD.

Figure S3. Wet weight, dry weight and water content. Skin (A) wet weight, (B) dry weight, and (C) water content in the different intervention groups. Skinned carcass (D) wet weight, (E) dry weight, and (F) water content in the different intervention groups. Total body (G) wet weight, (H) dry weight, and (I) water content in the different intervention groups.
